# What constitutes a high quality discharge summary? A comparison between the views of secondary and primary care doctors

**DOI:** 10.5116/ijme.538b.3c2e

**Published:** 2014-07-05

**Authors:** Rowan Yemm, Debi Bhattacharya, David Wright, Fiona Poland

**Affiliations:** 1 School of Pharmacy, University of East Anglia, UK; 2 School of Allied Health Professions, University of East Anglia, UK

**Keywords:** Patient discharge, discharge summary, patient transfer, interdisciplinary communication, medical education

## Abstract

**Objective:**

This study aimed to identify any differences in opinion between UK hospital junior doctors and community General Practitioners (GPs) with respect to the ideal content and characteristics of discharge summaries, and to explore junior doctors’ training for and awareness of post-discharge requirements of GPs.

**Methods:**

A piloted anonymous survey was posted to 74 junior doctors at a UK general hospital and 153 local GPs. Doctors were asked to rank discharge summary key content and characteristics in order of importance. GP discharge summary preferences and junior doctor training were also investigated. Non-respondents, identified by non-receipt of a separate participation card, were followed up once.

**Results:**

Thirty-six (49%) junior doctors and 42 (28%) GPs returned completed questionnaires. Accuracy was a priority with 24 (72%) GPs and 28 (88%) junior doctors ranking it most important. Details of medication changes were considered most important by 13 (39%) GPs and 4 (12%) junior doctors. Inadequate training in discharge summary writing was reported by 13 (36%) junior doctors.

**Conclusions:**

Although based on small sample sizes from one location, the level and range of differences in perceived importance of reporting medication changes suggests that many discharge summaries may not currently fulfil GP requirements for managing continuity of care. Results indicate that over a third of junior doctors felt inadequately prepared for writing discharge summaries. There may therefore be both a need and professional support for further training in discharge summary writing, requiring confirmatory research.

## Introduction

Accurate, comprehensive transfer of information about prescribed medicines across the healthcare interface is essential to ensure consistency between the treatment provided in hospital and in the community, and to ensure patient safety through the avoidance of medication-related inaccuracies. However, deficits in communication are widely reported.^1^^-^^4^ In 2009 a national survey of UK primary care General medical Practitioners (GPs) reported that they considered the information received on a discharge summary when a patient is transferred from secondary to primary care to be inadequate.^5^ They had particular concerns about discharge summary accuracy, timeliness and detail regarding medication changes.UK prescribing guidance, developed following extensive public consultation^6^ states that when patient care is transferred to the GP, secondary care doctors are obliged to provide details of the patient’s current and recent medicine use, medicine changes, length of intended treatment, monitoring requirements, and any new allergies or adverse reactions.^7^ Furthermore, in response to GPs raising concerns over receiving discharge information late, after the patient’s first post-discharge GP visit,^8^ a reduced timeframe of twenty-four hours after patient discharge for a discharge summary to be received in primary care was imposed from 2008.^9^ GPs have since reported an increased incidence of incomplete or inaccurate discharge information compromising patient safety.^10^In the UK, preparation of discharge summaries is primarily the responsibility of junior doctors, who are undertaking a two-year foundation training programme between graduating from medical school and undertaking specialist medical training. In 2009, a UK study reported that 90% of all discharge summary items were written by doctors in their first and second foundation year of training.^11^Although hospital consultants have overall responsibility for the discharge summaries produced, they will rarely check the content. However, a pharmacist carrying out a final check has been demonstrated to improve the quality of discharge summaries.^11^^,^^12^Both junior doctors and medical students have reported receiving inadequate guidance and training on how to write discharge summaries,^13^^,^^14^ and recognise that higher priority is often given to more immediately-pressing clinical tasks.^15^ Currently, each UK hospital uses its own unique prescribing system, and so, training of junior doctors in this area is difficult to standardise, with in-house training often being relied upon.Inadequacies in training for discharge summary preparation may be linked to limited intra-professional understanding between secondary and primary care doctors about discharge summary requirements, which could be a cause of poor quality summaries. The primary aim of this study was therefore to explore and compare the priorities and values of doctors working at either side of the primary and secondary care interface on medicines-related discharge information. Our objectives were to elicit what importance GPs give to the individual content, accuracy and timeliness of discharge summaries, and how their views compare to those of junior doctors.The secondary objectives were to investigate GP perceptions of and preferences for the timeliness, level of accuracy, pharmacy input and provision of medication changes on discharge summaries. We also investigated what training junior doctors had received on writing discharge summaries.

## Method

### Study design

A questionnaire survey to capture the opinions of both primary and secondary care doctors was undertaken following ethical approval from the University of East Anglia Faculty of Medicine and Health Ethics Committee.The study site in secondary care was a 600-bed general hospital in the UK which had been employing an electronic discharge system since 2008. This system enables discharge summaries to be generated electronically on the wards and emailed to the patient’s GP by the discharging doctor. Discharge summary content is typed manually into the electronic template. The primary care site was a group of 43 GP practices caring for 325,000 patients in the one UK region served largely by the study hospital.

### Participants and sample size calculation

At the time of study completion, 74 junior doctors were employed by the hospital and 173 GPs were located in the study site GP practices. Previous surveys to GPs and junior doctors have reported response rates of around 30%.^16^^-^^18^ An anticipated response rate of 30% would give a sample size of 46 GP surveys and 22 junior doctor surveys. For questions eliciting a response between 50% and 90%, these would provide 95% confidence intervals of 36% to 64% and 81% to 99% respectively for GPs, and 29% to 71% and 77% to 100% respectively for junior doctors. For questions eliciting a response between 50% and 90%, these would provide 95% confidence intervals of 36% to 64% and 81% to 99% respectively for GPs, and 29% to 71% and 77% to 100% respectively for junior doctors.

### Sampling methods

The survey was posted to all 153 GPs not involved in the piloting stage, and by internal mail to all 74 junior doctors employed by the hospital, together with a covering letter and survey participation card. Each doctor contacted was allocated a unique study reference code, which was printed on a separate survey participation card and sent to doctors alongside the survey. Receipt of a completed participation card indicated a response, thus preventing follow-up, whilst allowing survey answers to remain anonymous. A follow-up copy was sent to non-respondents after two weeks. Failure to respond to the second questionnaire after a further two weeks was treated as non-participation in the study.

### Data collection

The questionnaire was based on four characteristics and four types of content of discharge summaries. Selection of these characteristics and content was informed by the recommended minimum dataset of information to accompany a patient when they transfer between care settings^7^^,^^19^^,^^20^ and an audit conducted at the study hospital. This audit investigated the accuracy, pharmacy input, timeliness and quality of discharge summaries, and reported particularly poor adherence of summaries to the standards of providing information about medication changes and accurate medication-related information.^21^ Questions conforming to evidence based recommendations were prepared in order to collect both factual and attitudinal data from doctors.^22^The questionnaire was subsequently reviewed and refined in discussion with a multidisciplinary team comprising supervisory pharmacy practice researchers and a qualitative health researcher at UEA, a health economics researcher with specialist experience in questionnaire design, and senior clinical pharmacists and senior hospital doctors at the secondary care site in order to establish content validity.The questionnaire comprised three sections totalling 18 items and used a combination of Likert scale, and open responses as recommended for capturing attitudinal data, whilst the yes/no style was primarily used for factual data.^23^^,^^24^ Different versions were prepared for GPs and secondary care doctors.

From GPs, section 1 was designed to capture the following:

- Existing timeliness with which discharge summaries are received, and timeframe GPs considered acceptable for discharge summary receipt
- Current level of accuracy of discharge summaries and GP practice time spent resolving inaccuracies
- GP perceptions of the importance of a discharge summary being checked for accuracy prior to receipt
- From junior doctors, section 1 was designed to capture the following:-
- Frequency with which they wrote discharge summaries
- Frequency with which junior doctors reported discharge summaries as being checked for accuracy by a pharmacist before releasing to primary care
- Whether junior doctors had received formal training in discharge summary writing. If received, where this had taken place and perceived adequacy

Sections 2 and 3 were identical for both primary and secondary care doctors.Section 2 cited four characteristics of discharge summaries (timeliness, accuracy, completeness, and, spelling and grammar) and four items of discharge summary content (full list of medicines, medication changes, rationale for medication changes, and medication continuation plans), which doctors were asked to rank in order of importance on a Likert Scale of 1 to 4, where 1 is most important and 4 is least important. Doctors were asked to choose only one number per characteristic and content. This section contained a further three questions. The first two were open questions inviting comment on:

- Any discharge summary characteristics other than the four listed above perceived as important
- The one change most desired to existing discharge summaries produced at the secondary care study site

The last question in this section was a closed question asking respondents to identify whether details of medicines prescribed at discharge or details of medicine changes during hospitalisation are most important in a discharge summary.Section 3 asked for information from the respondent about their gender and number of years qualified as hospital or primary care doctor, so as to characterise the respondent sample.Content and face validity were further established through piloting the questionnaire with 20 randomly selected (using a list of GP reference numbers and a random number generator) GPs based in one UK region. The questionnaires were distributed by post therefore response rate was also estimated. Following piloting, ranking questions were changed, from asking respondents to assign a rank to each discharge summary component (characteristic or content), to asking respondents to draw a line between a list of ranks and components. This made it less likely that doctors would allocate more than one rank to each listed component. Pilot responses were excluded from the main analysis.

### Data analysis

Descriptive statistics were used to report doctors’ responses. Fisher’s exact test was used to compare the preferences of GPs and junior doctors for pharmacy checking and information provision on medication changes. Ranking choices were compared using Mann-Whitney U test. Simple thematic analysis was used to group and explore free text comments thus providing further depth to the quantitative data. Invalid responses, where the doctor had assigned more than one choice to each rank were excluded from the final analysis.

## Results

### Response rates

Of 232 questionnaires distributed (excluding the pilot), 36 (49%) junior doctors and 42 (28%) GPs returned a completed questionnaire.

### Training for junior doctors in writing discharge summaries

Twenty-eight (78%) junior doctors reported receiving formal training for writing discharge summaries, however only 6 (19%) received this as part of their medical degree, and 13 (36%) felt that the amount they received was inadequate. Mean (Standard Deviation) time spent preparing discharge summaries reported by junior doctors was 27% (19.2): 33% (22.5) for junior doctors in foundation year 1 and 19% (10.2) for junior doctors in foundation year 2.Six junior doctors raised the need for guidance and training on what information should be included on discharge summaries. All suggested this should be consultant or GP-led as represented by the following quote: “It would be helpful to hear directly from GPs what they need, and what information is useful/not useful to them” *(respondent 22).* Some specified that they would like this training to include guidance on appropriate content and that good practice examples would be helpful: “I would like to see some examples of what are considered good summaries” *(respondent 10)* and “some idea of content expectations would help” *(respondent 8).*

### Ranking questions

All 36 junior doctors answered both the ranking questions for the characteristics and content of discharge summaries, of which 33 (92%) and 32 (89%) responses respectively were valid (with only one rank assigned to each item). The same ranking questions were completed by 39 (93%) and 38 (91%) GPs respectively, of which 35 (90%) and 33 (87%) responses respectively were valid.

### Characteristics and content

Table 1 displays the average rankings assigned to the variables for characteristics and content of discharge summaries. The characteristic deemed most important by the greatest proportion of GPs and junior doctors was ‘accuracy’, which was assigned a rank of 1 (‘most important’) by 24 (73%) GPs and 28 (88%) junior doctors; no GPs or junior doctors ranked ‘accuracy’ as 4 (‘least important’). Only 3 (9%) GPs and no junior doctors ranked ‘timeliness’ as ‘most important’. The content deemed most important by the greatest proportion of GPs and junior doctors was details of ‘medication prescribed’, which was assigned a rank of 1 by 19 (54%) GPs and 23 (70%) junior doctors. ‘Medication changes’ were ranked as ‘most important’ by 13 (39%) GPs compared to 4 (12%) junior doctors. Statistically significant differences between the ranks assigned to medication changes and continuation plans by GPs and junior doctors were observed (Table 1).

**Table 1:** Median ranks assigned by doctors to discharge summary characteristics and content listed in the survey

Discharge summary component	Median (IQ) rank		Mann-Whitney U test
	GP	Junior Doctor	p value
Characteristics	n=33	n=32	
Accuracy	1 (1, 2)	1 (1, 1)	0.144
Completeness	3 (2, 4)	2 (2, 3)	0.006*
Timeliness	3 (2, 3)	3 (2, 3.75)	0.132
Grammar	3 (2, 4)	4 (3, 4)	0.068
Content	n=35	n=33	
Medication prescribed	1 (1, 3)	1 (1, 2)	0.078
Continuation plans	3 (2, 3)	3 (2, 4)	0.728
Medication changes	2 (1, 3)	3 (2, 3)	0.009*
Rationale for changes	3 (3, 4)	3 (3, 4)	0.812

*significance at the 0.05 level

GPs were largely dissatisfied with the quality of information about medication changes provided on discharge summaries, with 25 (61%) GPs describing details of changes as ‘poor’ or ‘very poor’. No GPs rated details of medication changes as ‘excellent’. Details of specific changes to medication, rather than a full list of all the prescribed medication, at discharge was preferred by 20 (49%) GPs compared to 10 (28%) junior doctors (Fisher’s Exact test, p=0.062).

### Accuracy of discharge summaries

The median (lower quartile, upper quartile) proportion of summaries which GPs reported to contain inaccuracies requiring practice time to address was 15 (10, 30) percent, with each inaccuracy taking a median (lower quartile, upper quartile) time of 0.5 (0.5, 1.0) hours to resolve. When considering accuracy checking of summaries by a pharmacist, 16 (44%) junior doctors reported not feeling comfortable with sending unchecked discharge information, whilst 29 (71%) GPs reported not feeling comfortable using unchecked discharge information to update their records (Fisher’s Exact test, p=0.023).A minority of GPs also reported being unaware that pharmacists sometimes do not check the discharge summary: “I had assumed that all the information we receive is checked for accuracy” *(GP respondent 22).* Others did not know where information about the pharmacy checking status could be found on the discharge summary: “I didn’t even notice the box which tells you if this has been done” *(GP respondent 21).*All GPs stated values of 24 hours or less for the ideal time in which to receive discharge summaries. However, 24 (59%) GPs would be willing to wait longer than 24 hours to receive a discharge summary in order to guarantee it had been checked for accuracy.

## Discussion

The present study investigated what importance doctors working at either side of the primary and secondary care interface gave to the individual content, accuracy and timeliness of discharge summaries. Results suggest that while there were some key differences in priorities between the two groups of doctors, the majority of both GP and junior doctor respondents considered accuracy to be the most important characteristic of discharge summaries.Whilst junior doctors appreciated the importance of discharge information being correct, in practice, a high error rate continues to be observed in discharge summaries.^5^^,^^25^^,^^26^ Recent research into the causes of prescribing errors by junior doctors at hospitals in the UK has shown that latent conditions (e.g. organisational processes, staffing), error-producing activities (e.g. busy environment, complex patient), active failures (e.g. mistakes) and lack of defences, such as a pharmacy check, can lead to errors being made.^27^ It may be that the environment within which doctors write summaries, the training and information resources available to them, and the possibility for human error introduced by them actually writing the summary, reduce means and time for accurate discharge summaries to be consistently prepared.

### Accuracy versus timeliness

Even though junior doctors reported considering accuracy as more important than timeliness, discharge summaries were often sent without being checked for accuracy by a pharmacist in order to expedite receipt by GPs and to meet the nationally agreed target for sending discharge information within 24 hours.^28^Whilst timely transfer of information is undoubtedly desirable, the rationale behind the 24 hour government target is unclear, as hospitals will generally supply at least seven days’ worth of medicines at discharge, and it is unlikely that a patient will need to visit their GP within 24 hours of being discharged. Currently, there is no UK evidence which supports implementation of the 24 hour target in terms of related patient outcomes. There is UK evidence, however, that improved continuity of care with GPs and structured discharge planning are effective in reducing emergency admissions and re-admissions respectively.^29^ GPs receiving inaccurate discharge information will disrupt continuity of care and structured discharge planning.Instead, this time target has placed increased pressure on junior doctors to send out discharge information, often for patients with whom they have had no experience of treating, and this may not allow sufficient time for a pharmacist to make a second check of the summary. Whilst junior doctors appear more content to comply, perhaps because their relative lack of experience may limit their understanding of the potential consequences or simply because of it being common practice, most GPs reported that they were not comfortable with using unchecked discharge information. The relative lack of concern expressed by junior doctors for checking the accuracy of summaries may indicate that they assume that GPs will always recognise whether or not the summary has been accuracy checked, or that the GP will provide a second check themselves upon receipt, rather than assuming that all the information provided on the summary is correct. Such assumptions would not be well-founded. GPs reported a preference for summaries to be received within 24 hours, but many would prefer to wait longer to ensure they had been checked for accuracy. A relaxation of the 24 hour target might therefore allow for improvements in the quality of summaries, which they would welcome.

### Medication changes

GPs have consistently been found to value provision of details of medication changes on discharge summaries, which validates the inclusion of this content in the Royal Pharmaceutical Society’s recent transfer of care guidance, as part of the minimum dataset recommended to be provided when care is transferred between settings.^19^GPs more often saw the explicit inclusion of details of medication changes more important than did the junior doctors. Nearly half of GPs preferred receiving only details of medication changes which had been made during the admission to receiving a full list of prescribed medication on discharge summaries, compared to just over a quarter of junior doctors who believed this to be the case. This key difference in priorities could indicate that junior doctors lack awareness of how GPs use information about medication changes for the purpose of updating the patient’s medication record after discharge. However, by ranking continuation plans higher than medication changes, junior doctors demonstrated an understanding of the need for care continuity post-discharge.Both GPs and junior doctors perceived details of the rationale for medication changes as least important. This may be because the rationale for changes made can sometimes be inferred from other discharge information provided, such as diagnosis. However, recent investigation into the documentation of prescribing decisions in a UK hospital found that hospital doctors are often unable to deduce why changes have occurred from the documentation available to them.^30^ Further research to explore how junior doctors gather information about medication, using the resources available to them, when composing discharge summaries is therefore warranted.

### Training and guidance

Reflecting findings from previous literature,^13^ junior doctors described having received little guidance on writing discharge summaries. Junior doctors expressed a desire for more training on the ideal content to include in a discharge summary, indicating a lack of confidence in what is required from them. This is consistent with recent findings from a study of postgraduate trainee medics in Canada, investigating trainees’ perceptions of their own and others’ roles at discharge, which found a lack of both inter and intra-professional clarity regarding roles and responsibilities. Substantial disagreement between trainees was reported for 38% of the 13 discharge roles described.^31^Inter-professional education has been introduced to UK undergraduate healthcare degree programmes and postgraduate courses, to foster “an understanding by every student of the roles of members of different professions in the health and social care team, with a view to ensuring that such teams work more effectively”,^32^ and is supported across UK nursing, medicine and pharmacy curricula.^32^^-^^34^ The concept of intra-professional education, however, which facilitates understanding of the roles of other workers within their own profession, is presently under-researched.^35^ In the present study, the GPs’ lack of awareness of the process and frequency by which summaries are checked for accuracy, combined with a lack of junior doctor confidence with respect to desirable summary content, suggests that promotion of intra-professional understanding between primary and secondary care doctors might assist in improving the quality of discharge summaries being produced. Further exploratory research in this area is therefore warranted.

### Study limitations

The present study was a small, local service evaluation of a UK general hospital and consequently may not be representative of all hospitals and the GP population they serve within the UK. GPs included were familiar with receiving electronically written and sent discharge summaries and so may have had different views to those using only paper-based summaries.For the purpose of this study only four different characteristics and content of discharge summaries were selected for examination. These had, however, been identified from existing guidance on the transfer of patient care and previous audit findings as being of relevance to the future research objectives of the team and secondary care organisation. Although doctors were asked to list any other components which they considered to be of importance, they were not asked to rank these additions. Some doctors stated that it was impossible to rank the content listed, and these respondents were excluded from final analysis. In instances where ranking is unsuitable, or where more information than simply a list of ranks is required, application of a Discrete Choice Experiment (DCE), a type of stated preference research in which a description of a service is provided according to its distinct specific properties, may be suitable. A DCE would, in the case of this study, enable the relative value of discharge summary components to be examined together with the willingness of doctors to trade between them in order to gain an increase or reduction in particular components.

## Conclusion

Although both GP and junior doctors identify accuracy as the most important characteristic of discharge summaries, junior doctors reported frequently sending information into primary care that had not been accuracy-checked by pharmacy, and worryingly, many seemed comfortable with doing so. The current 24 hour target allows only a minimal timeframe for hospital doctors to produce summaries, and only a narrow window of opportunity in which pharmacists can check summaries to ensure accuracy. Further research to investigate the appropriateness of any relaxation of the 24 hour target is therefore warranted.Junior doctors reported inadequate training and guidance for the preparation of discharge summaries and their ideal content, which might explain the difference observed between GPs and junior doctors’ perceptions of the importance of medication changes to be provided on summaries.One reason for this could be that junior doctors lack understanding of the GP’s role with respect to updating patients’ medication list following patients’ discharge from hospital. When prioritising work and deciding on the most appropriate actions, it is important to understand the perspective of people affected by those actions. If junior doctors’ perceptions of what is important in producing discharge information differ from those of general practitioners, then it is likely that problems will persist. Promotion of intra-professional understanding between the two groups of doctors through the provision of GP-led training might assist in bridging the gap between the two care settings and improving the quality of information produced by hospital doctors at discharge.

## 

### Conflict of Interest

The authors declare that they have no conflict of interest.
